# Biomarkers of TGF-β Signaling Pathway and Prognosis of Pancreatic Cancer

**DOI:** 10.1371/journal.pone.0085942

**Published:** 2014-01-20

**Authors:** Milind Javle, Yanan Li, Dongfeng Tan, Xiaoqun Dong, Ping Chang, Siddhartha Kar, Donghui Li

**Affiliations:** 1 Department of Gastrointestinal Medical Oncology, The University of Texas MD Anderson Cancer Center, Houston, Texas, United States of America; 2 Department of Pathology, The University of Texas MD Anderson Cancer Center, Houston, Texas, United States of America; University General Hospital of Heraklion and Laboratory of Tumor Cell Biology, School of Medicine, University of Crete, Greece

## Abstract

**Background:**

Transforming growth factor *(TGF)-β* signaling pathway, may act both as a tumor suppressor and as a tumor promoter in pancreatic cancer, depending on tumor stage and cellular context. TGF-β pathway has been under intensive investigation as a potential therapeutic target in the treatment of cancer. We hypothesized a correlation between TGF-βR2/SMAD4 expression in the tumor, plasma TGF-β1 ligand level, genetic variation in *TGF-B* pathway and prognosis of pancreatic cancer.

**Method:**

We examined TGF-βR2 and SMAD4 protein expression in biopsy or surgical samples from 91 patients with pancreatic ductal adenocarcinoma (PDAC) using immunohistochemistry. Plasma level of TGF-β1 was measured in 644 patients with PDAC using ELISA. Twenty-eight single nucleotide polymorphisms (SNP) of the *TGF-β1, TGF-β2, TGF-β3, TGF-βR1, TGF-βR2*, and *SMAD4* genes were determined in 1636 patients with PDAC using the Sequenom method. Correlation between protein expression in the tumor, plasma TGF-β1 level, and genotypes with overall survival (OS) was evaluated with Cox proportional regression models.

**Results:**

The expression level of TGF-βR2 and SMAD4 as an independent marker was not associated with OS. However, patients with both low nuclear staining of TGF-βR2 and high nuclear staining of SMAD4 may have better survival (*P* = 0.06). The mean and median level of TGF-β1 was 15.44 (SD: 10.99) and 12.61 (interquartile range: 8.31 to 19.04) ng/ml respectively. Patients with advanced disease and in the upper quartile range of TGF-β1 level had significantly reduced survival than those with low levels (*P* = 0.02). A significant association of *SMAD4* SNP rs113545983 with overall survival was observed (*P*<0.0001).

**Conclusion:**

Our data provides valuable baseline information regarding the TGF-β pathway in pancreatic cancer, which can be utilized in targeted therapy clinical trials. High TGF-β1 plasma level, *SMAD4* SNP or TGF-βR2/SMAD4 tumor protein expression may suggest a dependence on this pathway in patients with advanced pancreatic cancer.

## Introduction

Transforming growth factor-β (TGF-β) plays a vital role in cell cycle arrest, apoptosis, homeostasis, wound healing and immune regulation. In the case of cancers, TGF-β signaling plays a context-dependent dual role, both as a tumor suppressor in early stage disease and as a tumor promoter in established cancers [1]. There are three TGF-β isoforms, TGF-β1, 2 and 3. Of these, TGF-β1 is the most abundant in humans. TGF-β signaling occurs at several stages, starting with activation and release of the TGF-β1 followed by binding to three high affinity receptors (TGF-βR1, 2 and 3). TGF-βR1 and TGF-βR2 receptors dimerize after binding TGF-β at the cell surface [2]. These receptors, when sequentially activated phosphorylate a family of transcription factors, the SMADs. A recent exome sequencing study indicated that *TGF-βR2* is one of the 16 most commonly mutated genes in pancreatic cancer [3]. SMAD2 and SMAD3 are activated by TGF-βR1 and bind to the common partner SMAD4. SMAD6 and SMAD7 are inhibitory SMADs that block the phosphorylation of SMAD2 or SMAD3. The activated SMAD complex upon translocation to the nucleus regulates the transcription of several TGF-β-dependent genes that may have a context-dependent, tumor-suppressive or progressive role. Besides this ‘canonical’ TGF-β signaling pathway, there exist a variety of intracellular signaling pathways that are activated by TGF-β independently of SMAD2 or SMAD3 activation [4]. TGF-β signaling is activated in several known human cancers and is therefore an area of active investigation [5].

TGF-β pathway is one of the 12 core signaling pathways involved in pancreatic cancer [6]. Mutation in at least one of the TGF-β pathway genes occurs in 100% of the pancreatic tumors. LOH at 18q where SMAD4 gene is located occurs in 90% of pancreatic cancers while gene deletions and loss of protein expression occur in 50% [7], [8]. Loss of SMAD4 (DPC4) has been used to determine pancreatic origin in cases of metastases of unknown primary. It is believed that compromised TGF-β signaling may account for tumor progression rather than its initiation [4]. However, the actual role of SMAD4 in pancreatic cancer is still regarded as controversial. For instance, Biankin *et al* demonstrated that SMAD4 expression accounted for a worse prognosis in case of surgically resectable disease; patients with SMAD4 overexpression did not benefit from surgical resection in their study [9]. On the other hand, rapid autopsy data suggest that SMAD4 loss is associated with disseminated disease [10]. There are limited data regarding TGF-β receptor and SMAD4 expression or their prognostic significance in advanced pancreatic cancer patients. Furthermore, there are no data regarding TGF-β1 plasma level in pancreatic cancer and its correlation with prognosis.

Genetic variations of the TGF-β pathway genes have been reported in breast, ovarian, colon, non-small cell lung and colon cancers and may predict cancer susceptibility or have prognostic significance [11]–[15]. However, there are no data to our knowledge in regards to the same in pancreatic cancer. We hypothesize that TGF-β pathway activation is common in pancreatic cancer and genetic variations of the pathway, plasma TGF-β1 level and tumor TGF-βR2 or SMAD4 expression are associated with clinical outcome of pancreatic cancer. The identification of a cohort pancreatic cancer cases wherein the pathway is activated could potentially lead to patient selection for TGF-β-targeted therapy.

## Methods

### Patient Population and Biospecimens

All patients with pathologically confirmed pancreatic ductal adenocarcinoma (PDAC) and who signed an informed consent for medical record review and correlative studies for research were included. The Institutional Review Board of MD Anderson Cancer Center approved the study. Clinical information on date of patient diagnosis, date of death or last follow-up, tumor resection status, clinical tumor stage, and level of serum carbohydrate antigen 19–9 (CA19-9) at diagnosis was retrieved from patients medical records. Tissue samples, plasma and tumor DNA were retrieved from MD Anderson Tissue Banks.

### Immunohistochemistry

Immunohistochemistry was performed on formalin fixed paraffin embedded (FFPE) sections. The primary antibodies against TGF-βR2 (Novus Biologicals, LLC, Littleton, CO) and SMAD4 (Proteintech Group, Inc. Chicago, IL) were used at 1∶350 and 1∶450 dilutions, respectively. The antibody complex was detected using the ABC kit (Vector Laboratories, Burlingame, CA). Nuclear and cytoplasm staining was recorded for both markers. The staining intensity was scored as 0 for negative, 1 for weak, 2 for intermediate, and 3 for strong staining. The percentage of cells with positive staining were scored as 0 for none, 1 for 1–50%, and 2 for >50%. The final staining score was the product of the intensity and percentage scores. Each slide was evaluated by two investigators (Drs. Dongfeng Tan and Yanan Li) and consistent data from both evaluators were used in the final statistical analysis.

### Plasma Level of TGF-β1

Plasma level of TGF-β1 was measured by the Immune Monitoring Core Laboratory of MD Anderson using the MSD® 96-Well MULTI-ARRAY®Human TGF-β1 Assay kit (Meso Scale Discovery, Rockville, MD). All samples were analyzed in duplicate and each assay had a positive and a negative control. The variance of the duplicate samples was less than 10%. All patients involved in this assay were recruited to a case control study at MD Anderson Cancer Center [16], [17]. Blood samples were collected before the cancer treatment or at the time of diagnosis in 95% of the cases. Plasma samples had been stored at −80°C without thawing before use in this assay.

### Genotyping

DNA was extracted from peripheral lymphocytes in the majority of the samples and from FFPE in 27 samples. Genotyping used the Sequenom method as previously described [18]. A total of 28 SNPs of the *TGF-β1*, *TGF-β2*, *TGF-β3*, *TGF-βR1*, *TGF-βR2* and *SMAD4* genes were selected with a focus on potentially functional SNPs, i.e. SNPs in the coding region (nonsynonymous or synonymous), untranslated region (UTR), promoter region and splicing sites, or ins/del and frame-shift SNPs. SNPs were identified from the NCBI SNP database and SNP500 Cancer database or via literature review and functional analysis using the F-SNP software (http://compbio.cs.queensu.ca/F-SNP/). About 10% of the samples were analyzed in duplicate and inconsistent data were excluded from the final statistical analysis.

### Statistical Analysis

IHC score and plasma TGF-β1 levels were presented as mean ± standard deviation. The mean difference between groups was compared using the Student’s t-test. The associations of these markers and OS were analyzed using Kaplan Meier plot, log-rank test and Cox proportional hazards models with adjustment for sex, race, age, tumor stage, and CA19-9 levels. These markers were also analyzed as categorical variables using the median or quartiles as cutoff values.

The distribution of genotypes was examined for Hardy-Weinberg equilibrium with the goodness-of-fit chi-squared test. Genotype and allele frequency of the SNPs were determined by direct gene counting. The homozygous and heterozygous genotypes were combined if the frequency of the homozygote was very low or if both genotypes had the same trend of effect [e.g., shorter overall survival (OS) compared with the referent group]. The association between genotype and OS was estimated using the Kaplan-Meier plot and log-rank test. Hazard ratios (HR) and 95% confidence interval (CI) were estimated using the multivariate Cox regression proportional hazards models. All statistical testing used SPSS software (SPSS Inc, Chicago, IL). *P* value of <0.05 was considered statistically significant. False positive finding associated with multiple testing was controlled by Bonfferoni correction.

## Results

The study population was identified from a case-control study on pancreatic cancer conducted at The University of Texas MD Anderson Cancer Center from 1999 through 2012 [16], [17]. Three groups of patients were identified: 1) 120 patients had adequate biopsy or surgical samples for the immunohistochemistry; 2) 644 patients had blood samples collected for the plasma TGF-β1 measurement; and 3) 1636 patients had adequate DNA samples available for genotyping. The demographic and clinical characteristics of the three study populations are described in Table 1. The age, gender and racial/ethnic distributions of the patients were representative of the MD Anderson patient population. The mean age is 60.6, 61.4 and 62.1 years for patients included in the IHC, ELISA and genotyping study, respectively. Men consisted 72.5%, 61.5% and 59.0% of the three study populations. More than 85% of the study subjects were non-Hispanic whites. Nearly 74% of the patients in IHC group had metastatic disease and therefore the tissue samples used for IHC were mostly biopsy samples [Stage II: 8 (8.8%); Stage III: 16 (17.6%); Stage IV: 67 (73.6%)]. Information on tumor grade was available in 60 samples of which, 20 were moderately differentiated and 40 were poorly differentiated tumors.

**Table 1:** Characteristics of the patient population (n, %).

Variable	IHC	Plasma	Genotyping
	(n = 91)	(n = 644)	(n = 1,636)
Age (years)			
<50	14 (15.4)	98 (15.2)	218 (13.3)
51–60	28 (30.8)	184 (28.6)	459 (28.1)
61–70	37 (40.7)	237 (36.8)	597 (36.5)
>70	12 (13.2)	125 (19.74)	341 (20.8)
Race			
White	71 (84.6)	629 (97.7)	1,429 (87.3)
Hispanic	1 (1.1)	4 (0.6)	87 (5.3)
Black	10 (11.0)	8 (1.2)	91 (5.6)
Others	3 (3.3)	2 (0.4)	29 (1.8)
Sex			
Male	66 (72.5)	396 (61.5)	965 (59.0)
Female	25 (27.5)	248 (38.5)	671 (41.0)
Stage			
Localized	8 (8.8)	162 (25.2)	533 (32.6)
Locally advanced	16 (17.6)	197 (30.6)	504 (30.8)
Metastatic	67 (73.6)	285 (44.3)	599 (36.6)
CA19-9 (Unit/mL)			
<47	13 (14.3)	117 (18.2)	340 (20.8)
48–500	19 (20.9)	231 (35.9)	612 (37.4)
501–1000	11 (12.1)	60 (9.3)	158 (9.7)
>1000	34 (37.4)	207 (32.1)	435 (26.6)
Missing	14 (15.4)	29 (4.5)	91 (5.6)

### Protein Expression

**Figure 1:**
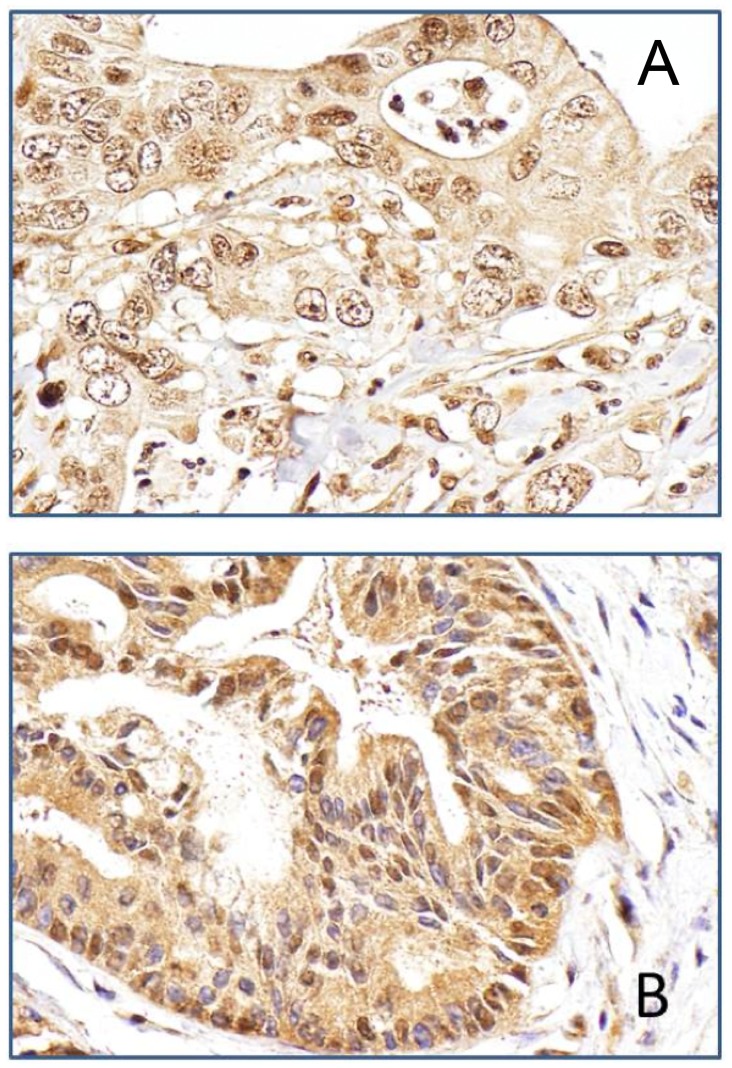
Typical Immunohistochemical staining pattern for TGF-βR2 and SMAD4 in pancreatic adenocarcinoma tumor tissues. A: Positive nuclear expression of TGF-βR2 in a moderately differentiated ductal adenocarcinoma of the pancreas (Magnification: 10×40). B: Positive nuclear expression of SMAD4 in moderately differentiated ductal adenocarcinoma of the pancreas (Magnification: 10×40).

**Figure 2:**
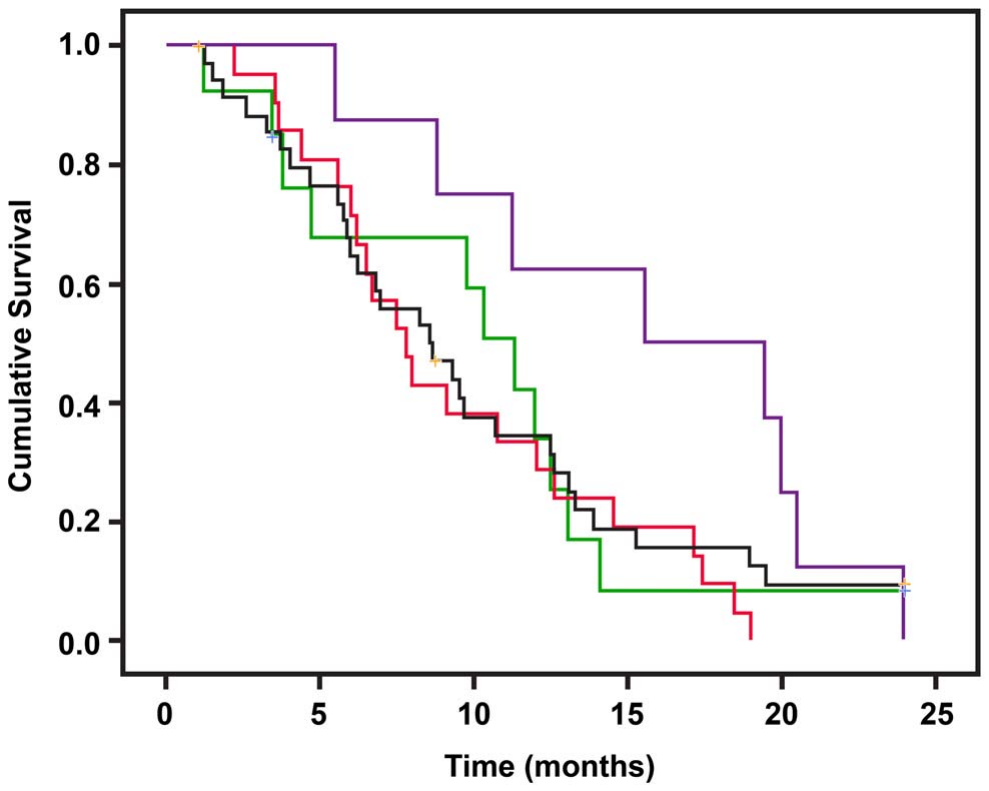
Overall survival of patients with various nuclear expression levels of TGF-βR2 and SMAD4. Nuclear staining score 0–1 is defined as low for TGF-βR2 and a score 0 as low for SMAD4. Red line: TGF-βR2 is high and SMAD4 is low (HL); black line: both high (HH); green line: both are low (LL); purple line: TGF-βR2 is low and SMAD4 is high (LH). The median survival times are 7.8, 8.6, 11.3, and 15.6 months for the HL, HH, LL and LH groups, respectively. Log-rank test P values and results of Cox regression analysis are presented in Table 2. Thus, TGFβ-R2/SMAD4 ratio may be prognostic, with low values corresponding with an improved survival.

A total of 91 samples were stained for TGF-βR2 and SMAD4. Quantitative evaluation was achieved in 88 samples for TGF-βR2 and 81 samples for SMAD4 (Fig. 1). Nuclear and cytoplasm staining was observed, respectively, in 81 (92%) and 87 (99%) samples for TGF-βR2 and in 47 (58%) and 72 (89%) samples for SMAD4 (Fig. 1). The overall IHC score for either marker was not associated with OS (data not shown). Patients with a higher nuclear staining score for TGF-βR2 had a relatively shorter OS than those with a lower score (Median survival time [MST] 12.0 versus 8.6 months, Table 2), but this difference was not statistically significant. Nuclear staining of SMAD4 was present more frequently (16/20, 80%) in poorly differentiated tumors than in moderately differentiated tumors (19/40, 47.5%) (*P* = 0.016, χ^2^ test). Furthermore, when TGF-βR2 and SMAD4 nuclear expression was analyzed in combination, we noted that patients with low expression of TGF-βR2 and high expression of SMAD4 had a significantly longer OS than others (Fig. 2), although this difference was not statistically significant after adjusting for other clinical predictors (Table 2).

**Table 2:** Nuclear staining for TGF-βR2 and SMAD4 and association with overall survival.

Marker		No. of Patients	No. of Death	MST (months)	*P*_log-rank_	HR (95% CI)	*P* value
TGF-βR2							
Low (score 0–1)		24	21	12.0		1.0	
High (score 2)		64	51	8.6	0.135	1.54 (0.88–2.68)	0.131
SMAD4							
Low (score 0)		34	32	9.1		1.0	
High (score 1–2)		47	40	9.3	0.223	0.84 (0.51–1.37)	0.480
TGF-βR2	SMAD4						
High	Low	21	21	7.8		1.0	
High	High	36	30	8.6	0.767	0.66 (0.31–1.41)	0.818
Low	Low	13	11	11.3	0.532	0.76 (0.38–1.52)	0.939
Low	High	8	8	15.6	0.007	0.40 (0.15–1.06)	0.062

MST: median survival time; HR: hazard ratio; CI: confidence interval.

HR was adjusted for age, sex, race, stage and CA19-9.

### Plasma TGF-β1 Level

Plasma level of TGF-β1 was measured in 644 patients. The mean and median level of TGF-β1 was 15.44 (SD 10.99) and 12.61 (interquartile range: 8.31 to 19.04) ng/ml, respectively. The level of TGF-β1 was relatively higher in patients with localized tumor than those with advanced tumors. The mean ± SD of TGF-β1 level was 17.2±14.3, 13.9±8.7 and 15.5±10.0 ng/ml in patients with localized, locally advanced and metastatic tumors, respectively (*P* = 0.02, ANOVA). However, the level of TGF-β1 was not associated with OS in patients with localized tumor (Table 3) or in the entire study population (MST = 12.9 and 11.1 months for those in the lower quartiles versus those in the upper quartile range, *P* = 0.78, log-rank test). However, patients with locally advanced or metastatic disease and in the upper quartile range of TGF-β1 level had significantly reduced survival than their counterparts (Table 3). TGF-β1 remained as a significant predictor in a Cox regression model with adjustment for demographics and other clinical factors.

**Table 3:** Plasma TGF-β1 level and overall survival.

Patients TGF-β1 (ng/ml)	No. of Patients	No. of Deaths	MST (months)	P value (log-rank)	HR (95% CI)	P value
Localized				0.632		0.548
<19.05	109	78	26.43		1.0	
≥19.05	53	35	27.66		0.88 (0.58–1.33)	
Locally advanced & metastatic				0.006		0.011
<19.05	375	308	10.67		1.0	
≥19.05	107	101	7.97		1.35 (1.07–1.69)	

19.05 is the 25^th^ percentile of the TGF-β1 value of the entire study population.

HR was adjusted for age, sex, race, stage and CA19-9.

### Genotyping

**Figure 3:**
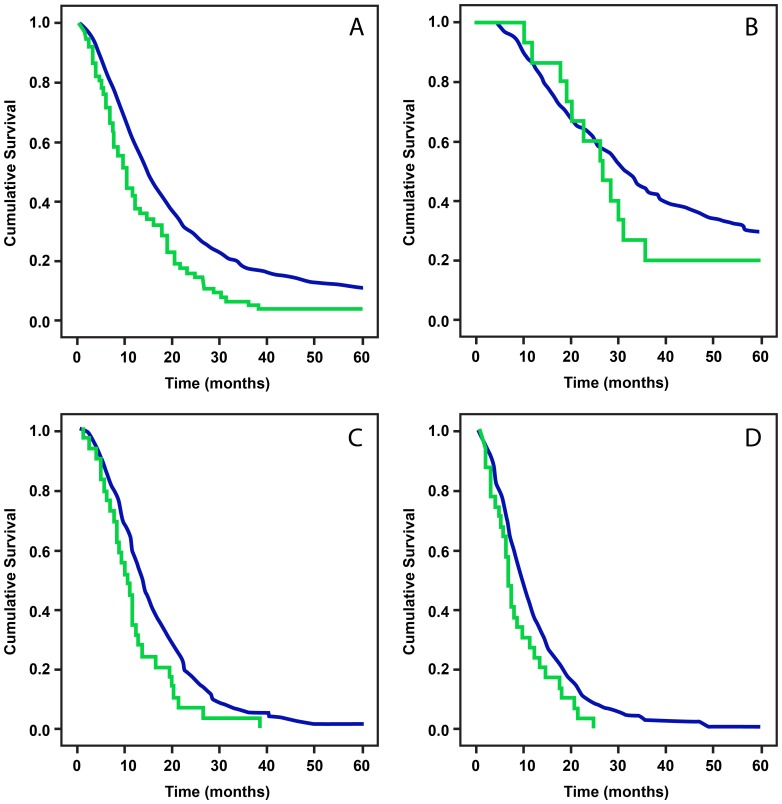
Plot of overall survival curve in patients with all patients (A), patients with localized (B), locally advanced (C) or metastatic tumors by SMAD4 SNP rs2704733 genotype. Blue line: AA genotype; green line: AG/GG genotype. AA genotype was associated with an improved survival in the entire study population. On subgroup analysis, this survival difference was more relevant for advanced disease stage. P values by log-rank test are <0.0001, 0.369, 0.007, and 0.031 for panels A, B, C and D, respectively.

Genotyping was performed in 1636 patients. The reported minor allele frequency and potential functional significance of the 28 tested SNPs is described in Table 4. No variant allele was detected for nine SNPs. Among the six common SNPs with minor allele frequency greater than 5%, three followed the Hardy Weinberg Equilibrium (HWE) and three deviated from the HWE (Table 2). The genotype distribution and overall survival time by genotype of the 19 informative SNPs are presented in Table 5. A significant association of *SMAD4* SNP rs113545983 with OS was observed (Panel A, Fig. 3), and the association was stronger in patients with advanced disease (Panels C and D, Fig. 3) than those with localized disease (Panel B, Fig. 3). The mutant G allele of SNP rs113545983 remained as a significant predictor for death in Cox regression model after adjusting for stage and resection status among all patients (HR: 1.54, 95% CI: 1.21–1.96, *P*<0.001). No other SNPs showed significant association with OS. TGF-βR2 SNP rs2248048 had a weak association with OS without statistical significance (*P* = 0.09, log rank test).

**Table 4:** MAF and functional significance of the SNPs investigated in the current study.

Gene	SNP ID	Allele	Functional	MAF
***TGF-β*** *1*	rs35383147	A/G/T	ThR24 =	NA
***TGF-β*** *2*	rs6684205	G/A	intron	0.482
	rs114663618*	C/T	intron	NA
	rs10482810	G/C	Val235Leu	0.001
	rs7531245	A/C	intron	0.011
***TGF-β*** *3*	rs11466415	C/T	Ala13 =	0.002
	rs4252315	C/T	Thr60Met	0.003
***TGF-β*** *R1*	rs11466445*	−/GGCGGCGGC	Ala23fs	NA
	rs75857126*	C/T	Ile147Thr	NA
	rs113982335*	A/C	Asn160His	NA
***TGF-β*** *R2*	rs2043136	T/C	3′ near gene	0.314
	rs1155705	A/G	intron	0.425
	rs3087465	A/G/T	5′ near gene	0.337
	rs11466530*	C/T	intron	0.001
	rs17026332	A/C	3′ UTR	0.009
	rs11466512	A/T	intron	0.311
	rs61762550*	C/T	Arg218Trp	0.0005
	rs114457508	T/G	Val276Gly	NA
	rs28934568*	C/T	Leu333Pro	NA
	rs34833812	C/T	Thr340Met	0.006
	rs3209742*	A/T	Glu341Val	NA
	rs17854016	G/T	Glu360Ter	NA
	rs2228048	C/T	Asn41 =	0.083
*SMAD4*	rs4939650	C/G	5′ near gene	0.497
	rs1801250	C/T	Phe362 =	0.002
	rs112891188*	C/T	Ala220 =	NA
	rs75667697	G/T	Leu229Arg	NA
	rs113545983	A/G	Gln549 =	NA

MAF: minor allele frequency.

No variant allele was detected in the current study population.

“ = ” represents synonymous gene variants, i.e. no amino acid change.

**Table 5:** Genotype frequency and median survival time.

Gene	RS#	Genotype	N	MST	*P*
***TGF-β1***	rs35383147	G	1618	14.6	0.26
		GT/T (8/1)	9	14.6	
***TGF-β2***	rs6684205	A	557	15.1	0.9
		AG	858	14.6	
		G	155	14.9	
	rs10482810	G	1618	14.6	0.24
		GC	18	14.8	
	rs7531245	A	1561	14.7	0.61
		AC	14	16.1	
***TGF-β3***	rs11466415	C	1589	14.7	0.59
		CT	47	12.8	
	rs4252315	C	1629	14.6	0.37
		CT	2	5.5	
***TGF-βR2***	rs1155705	A	751	14.9	0.9
		AG	723	14.4	
		G	159	13.9	
	rs2043136*	T	876	14.9	0.82
		TC	605	14.5	
		C	122	14.1	
	rs3087465*	G	929	14.5	0.96
		GA	592	14.8	
		A	103	14.3	
	rs11466512*	T	802	15	0.4
		AT	702	13.9	
		A	129	14.7	
	rs114457508	T	1629	14.7	0.34
		TG	3	7.8	
	rs34833812	C	1565	14.8	0.09
		TC/T (3/1)	4	22.5	
	rs17854016	G	1631	14.7	0.97
		GT	1	20.4	
	rs2228048	C	1572	14.6	0.09
		CT/T (56/2)	58	17.6	
	rs17026332	C	1557	14.7	0.62
		CA/A	47	19.1	
***SMAD4***	rs4939650	C	519	15.1	0.95
		CG	847	14.4	
		G	260	14.6	
	rs1801250	T	1621	14.7	0.73
		CT/C (3/1)	4	6.4	
	rs75667697	T	1631	14.7	0.28
		GT	3	10.5	
	rs113545983	A	1543	14.8	**<.0001**
		AG	55	10.2	
		G	24	10.7	

SNPs that are conformed in HWE.

## Discussion

Our goal in the current study was to investigate biomarkers in the TGF-β pathway that could have prognostic value and potentially predictive value for targeted therapy with inhibitors. We interrogated the tumor biorepository at MD Anderson Cancer Center and examined archival material including DNA, plasma and tumor tissue samples for SNPs, TGF-β1 plasma level and protein expression of TGF-βR2 and SMAD4. We observed that patients with low expression of TGF-βR2 and high expression of SMAD4 in their tumors had a significantly longer OS than other subgroups in our study. We also noticed that patients with advanced disease and high TGF-β1 plasma level had significantly reduced survival than those with a lower level of TGF-β1. Finally, we detected a significant association of *SMAD4* SNP rs113545983 with patient survival. These observations provide valuable baseline information regarding the TGF-β signaling pathway in pancreatic cancer, which can be utilized in future targeted therapy clinical trials. The TGF-β signaling pathway includes the ligands and the receptors; and the ligand-receptor interactions lead to signal transduction via SMADs. Previous IHC analysis has shown the presence of ligand TGF-β1, TGF-β2 and TGF-β3 in PDAC cancer cells and the presence of TGF-β2 was associated with advanced tumor stage [19]. TGF-βR2 mRNA was expressed in the majority of cancer cells and enhanced levels of TGF-βR2 has been suggested to have a role in regulating human pancreatic cancer cell growth [20]. *TGF-β2R* and *SMAD4* gene was mutated in 4% and 50% of the human PDAC, respectively [21]. Lack of SMAD4 expression in the tumor has been associated with more aggressive disease [22]. There are very limited data investigating the prognostic value of TGF-β pathway proteins in advanced pancreatic cancer patients. Although we did not find a significant correlation between SMAD4 and TGF-βR2 expression individually with prognosis, we observed that TGF-β2R and SMAD4 protein expression was detected in 81% and 47% of the tumors and patients with low expression of TGF-βR2 and high expression of SMAD4 had a significantly longer OS than other subgroups in our study. Hua et al, reported similar findings in a smaller retrospective study of patients with advanced pancreatic cancer [23]. A prior study from our institution suggested that SMAD4 loss correlates with an adverse outcome in patients with locally advanced pancreatic cancer receiving gemcitabine, oxaliplatin and cetuximab [24]. As mentioned earlier, SMAD4 loss was associated with disease dissemination in locally advanced and metastatic pancreatic cancer cases by Iacobuzio-Donahue, et al in an autopsy series [10]. On the other hand, a literature-based meta-analysis of biomarkers in operated pancreatic cancer did not support the prognostic value of SMAD4 in surgically resectable disease [25]. These findings are consistent with a stage-dependent role of the TGF-β pathway.

Furthermore, we found that patients with advanced disease and high TGF-β1 plasma level had significantly reduced survival than those with a lower TGF-β1 level. This association was not seen in patients with localized disease. A previous study has shown that lack of TGF-β1 expression in tumor tissues was associated with increased postoperative survival [19]. The reason for the association between high TGF-β1 plasma level and poor survival at advanced disease stage (and not with early stage disease) is unclear. However, these results are consistent with preclinical data that suggest that loss of the growth inhibitory response to TGF-β signaling varies directly with the malignant stage of the tumor and the more aggressive forms actually switch to autocrine and/or paracrine growth stimulated by TGF-β. Tumors can also secrete TGF-β1 leading to altered anti-tumor immunity [26], [27]. To our knowledge, there are no prior data investigating the prognostic role of TGF-β1 plasma level in pancreatic cancer. Prior studies in breast, prostate, esophageal and bladder cancer indicate a correlation between high TGF-β1 plasma level and poor prognosis [28]–[31]. Consistency with these reports and the relatively large sample size of our study adds to the strength of our findings. Because of the challenges in accessing the target tissues in patients with advanced disease, plasma biomarkers for the TGF-β1 pathway may have a greater clinical value.

Genetic variations of the TGF-β pathway have been linked to prognosis and survival in lung cancer by another study conducted at our institution. In patients receiving platinum-based chemotherapy; BMP2:rs235756 and SMAD3:rs4776342 were significantly associated with survival [15]. There are however, no studies in pancreatic cancer correlating genomic variations of this pathway with prognosis. We noted a significant association between *SMAD4* SNP rs113545983, a synonymous SNP, with OS in this study. Patients carrying the variant allele had significantly shorter OS and increased risk of death after adjusting for other clinical predictors. This SNP is a synonymous SNP that does not produce altered coding sequences and amino acid substitution. However, a previous study has demonstrated that a synonymous SNP could result in a protein product with altered drug and inhibitor interactions 32. Thus, the functional consequence by this SNP needs to be investigated. It is also possible that this SNP is in linkage disequilibrium with other functional SNPs of this gene or some important genes in this chromosome location. Further investigations will help to determine how this SNP is functionally associated with the SMAD4 signaling transduction and survival in pancreatic cancer patients.

The study has several limitations. Because of the limitation of tissue samples and the low MAF SNP selected in the study, we could not examine the correlation between genotypes, plasma marker level and tissue markers. We only measured a few selected markers from the many molecules that are involved in this complex signaling pathway. Nevertheless, our data provide valuable baseline information regarding this pathway expression in pancreatic cancer, which can be utilized in targeted therapy clinical trials. Based on the above findings, it can be hypothesized that detection of high TGF-β1 plasma level, SMAD4SNP rs113545983 or high TGF-βR2/SMAD4 tumor protein expression ratio may suggest a dependence on this pathway in patients with advanced pancreatic cancer and this subset may potentially benefit from TGF-β-targeted therapy.
